# Presence of hepatitis E virus in testis of naturally infected wild boars

**DOI:** 10.1111/tbed.14682

**Published:** 2022-09-01

**Authors:** María A. Risalde, Mario Frias, Javier Caballero‐Gómez, Pedro Lopez‐Lopez, Christine Fast, Saúl Jiménez‐Ruiz, Irene Agulló‐Ros, Martin Eiden, Débora Jiménez‐Martín, Ignacio García‐Bocanegra, Antonio Rivero, José Carlos Gómez Villamandos, Antonio Rivero‐Juarez

**Affiliations:** ^1^ Departamento de Anatomía y Anatomía Patológica Comparadas y Toxicología Grupo GISAZ UIC Zoonosis y Enfermedades Emergentes ENZOEM Universidad de Córdoba Campus de Rabanales Edificio Sanidad Animal Córdoba Spain; ^2^ Unidad de Enfermedades Infecciosas Grupo de Virología Clínica y Zoonosis Instituto Maimónides de Investigación Biomédica de Córdoba (IMIBIC) Hospital Reina Sofía Universidad de Córdoba (UCO) Córdoba Spain; ^3^ CIBERINFEC ISCIII – CIBER de Enfermedades Infecciosas Instituto de Salud Carlos III Spain; ^4^ Departamento de Sanidad Animal Grupo GISAZ UIC Zoonosis y Enfermedades Emergentes ENZOEM Universidad de Córdoba Campus de Rabanales Edificio Sanidad animal Córdoba Spain; ^5^ Institute of Novel and Emerging Infectious Diseases Friedrich‐Loeffler‐Institut, Greifswald‐Insel, Riems Germany; ^6^ Grupo Sanidad y Biotecnología (SaBio), Instituto de Investigación en Recursos Cinegéticos IREC (UCLM‐CSIC‐JCCM), Universidad de Castilla‐la Mancha (UCLM) Ciudad Real Spain

**Keywords:** genotype 3, hepatitis E virus, testicle, wild boar

## Abstract

The hepatitis E virus (HEV) is the main cause of viral acute hepatitis in the world, affecting more than 20 million people annually. During the acute phase of infection, HEV can be detected in various body fluids, which has a significant impact in terms of transmission, diagnosis or extrahepatic manifestations. Several studies have isolated HEV in the genitourinary tract of humans and animals, which could have important clinical and epidemiological implications. So, our main objective was to evaluate the presence of HEV in testis of naturally infected wild boars (*Sus scrofa*). For it, blood, liver, hepatic lymph node and testicle samples were collected from 191 male wild boars. The presence of HEV was evaluated in serum by PCR, as well as in tissues by PCR and immunohistochemistry. Four animals (2.09%; 95%CI: 0.82–5.26) showed detectable HEV RNA in serum, being confirmed the presence of HEV‐3f genotype in three of them by phylogenetic analysis. HEV was also detected in liver and/or hepatic lymph nodes of the four animals by RT‐PCR, as well as by immunohistochemistry analysis. Only one of these wild boars also showed detectable viral load in testis, observing HEV‐specific labelling in a small number of fibroblasts and some Sertoli cells. Our results confirm the presence of HEV genotype 3 in naturally infected wild boar testis, although no associated tissue damage was evidenced. This study does not allow us to discard semen as a possible source of HEV transmission in suids. Future experimental studies are necessary to evaluate the impact of HEV genotype 3 on fertility and the possibility of transmission through sexual contact in this specie.

## 1 INTRODUCTION

Hepatitis E virus (HEV) belongs to the genus Orthohepevirus (family Hepeviridae), including *Paslahepevirus balayani* species the most important strains for human and animal health (Purdy et al., 2022). HEV is the main cause of viral acute hepatitis in the world, affecting more than 20 million people annually (WHO, 2017).

Eight genotypes (1 to 8) of HEV have been described, of which only genotypes 1 and 2 exclusively affect humans and are associated with typical epidemics of emerging countries, while the genotypes 3 and 4 have been isolated in a wide range of animals and are the main cause of sporadic autochthonous cases of hepatitis E in industrialized countries (Denner, 2019; Meng, 2013; Nimgaonkar et al., 2018). Here, hepatitis caused by HEV is endemic with the appearance of small sporadic outbreaks (Purcell & Emerson, 2008), where the liver is the main affected organ, although other extrahepatic manifestations have also been observed (Horvatits & Pischke, 2018). Transmission of genotypes 3 and 4 is mainly zoonotic, through direct contact or consumption of raw or undercooked pork or game meat products or shellfish (Faber et al., 2018; Syed et al., 2018; Wang et al., 2019); nevertheless, other forms of contagion have also been demonstrated, such as blood transfusions and organ transplantation (Domanović et al., 2017; McPherson et al., 2018). Although these sources of transmission have been well documented in different studies and are considered the main widespread routes, other possible transmission routes have also been suggested.

The efficiency of sexually transmitted HEV infection and its impact on fertility is unknown. There are only few reports on the presence of HEV RNA in human semen (Huang et al., 2018; Li et al., 2019; Soomro et al., 2017) and in reproductive organs of mice and suids experimentally infected (Schlosser et al., 2014; Situ et al., 2020). However, in the animal experimental cases, the infection was established intravenously, where distribution of the virus is always greater. Furthermore, histological analysis revealed damage on testicular tissue in humans and mice, suggesting that viral replication is possible at this anatomical site (Huang et al., 2018; Situ et al., 2020). Nevertheless, in these studies the HEV genotype involved was genotype 4 (mainly present in Asia). Currently, there is no evidence of viral infection in the reproductive organs of naturally infected animals and there are no data supporting this finding in the case of genotypes with global distribution, such as genotype 3. Therefore, our objective was to evaluate the presence of HEV in testis of naturally infected wild boars (*Sus scrofa*).

## 2 MATERIALS AND METHODS

### 2.1 Study design and sampling strategy

A total of 194 male wild boar were randomly sampled in 32 hunting areas in Andalusia, southern Spain (36°N–38°600 N, 1°750 W–7°250 W), and Extremadura, southwestern Spain (39°49 N–38°54 N, 5°11 W–6°20 W) during the hunting seasons (from 15 October to 15 February) from 2017/2018 to 2020/2021. Age was determined on the basis of tooth eruption and animals under 12 months old were classified as juveniles, those between 12 and 24 months as subadults and those over 2 years old as adults.

At post‐mortem examination carried out at the meat board, a whole blood sample was taken from all the animals using puncture of the cavernous sinus of the dura mater (Arenas‐Montes et al., 2013). Blood samples were centrifuged at 700 *g* for 10 min to obtain the serum, which was frozen at –80°C until analysis. Samples of liver, hepatic lymph nodes and testis were also collected from all the animals. The tissue samples were divided in two, a piece of each was submerged in RNAlater^®^ Stabilization Solution (Thermo Fisher Scientific Inc., Waltham, MA, USA) and frozen at –80°C until analysis, and the other piece was fixed by immersion in 10% buffered formalin solution for histopathological and immunohistochemical study.

### 2.2 Molecular evaluation of HEV

RNA was extracted from 200 μl of serum using the commercial QIAamp MinElute Virus Spin Kit (QIAgen, Hilden, Germany) and an automated procedure (QIAcube. QIAgen, Hilden, Germany). Tissue RNA (liver, lymph node and testis) was extracted from 30 mg of the samples using the commercial RNeasy Mini Kit (QIAgen, Hilden, Germany). The purified RNA was eluted in a total volume of 50 μl.

RT‐PCR was performed with the CFX Connect real‐time PCR system (Biorad, Hercules, California, USA), following the protocol previously described and especially designed for *Orthohepevirus* A detection (Frías et al., 2021). For the reaction, the commercial kit QIAgen One‐Step PCR Kit (QIAgen, Hilden, Germany) was used. The final volume of the PCR was 50 μl, including a positive (WHO standard virus; provided by Paul‐Ehrlich‐Institut laboratory, Langen, Germany; Code 6329/10) and a negative control (water). Detection limit of the PCR was set at 21.86 IU/ml (CI_95%_: 17.38–34.30 IU/ml) (Frías et al., 2021).

For genotyping analysis, nested RT‐PCR of the ORF1 region was carried out following the protocol described elsewhere (Johne et al., 2010). The second amplification product of 334 bp was sequenced using the BigDye Terminator Cycle Sequencing Ready Reaction Kit on an ABI PRISM 3100 Genetic Analyzer (Applied Biosystems, Foster City, CA, USA). The consensus sequence was obtained using SeqMan NGen^®^ program (version 12.0, DNASTAR, Madison, WI). For subtype assignment and phylogenetic analysis, the HEVnet genotyping tool was used (https://www.rivm.nl/mpf/typingtool/hev/), then confirmed by BLAST.

### 2.3 Histopathological and immunohistochemical examination of tissues for HEV

The formalin‐fixed samples were dehydrated with a graded series of alcohols, cleared in xylene and embedded in paraffin wax. After making 4 μm sections and staining with haematoxylin and eosin (H&E), two blinded and experienced observers then performed a histopathological evaluation of the tissue sections.

Sections of the formalin‐fixed paraffin‐embedded tissue samples (3 μm) were routinely processed for immunohistochemistry (IHC) using the avidin‐biotin‐peroxidase complex (ABC) method described by Risalde et al. (2017). Briefly, endogenous peroxidase activity was exhausted by incubation of the samples with 0.3% hydrogen peroxide in methanol for 30 min at room temperature (RT, approx. 25°C). For antigenic unmasking, the sections were incubated with 0.2% proteinase K (Sigma‐Aldrich, St. Louis, Missouri, USA) in 0.05 M Tris‐buffered saline (TBS; pH 7.6) for 8 min at 37°C. Then, sections were covered with 20% normal goat serum (Vector Laboratories, Burlingame, CA) in 0.01 M phosphate‐buffered saline (PBS) at RT for 30 min. After blocking, the sections were incubated with a rabbit anti‐HEV gt3 hyperimmune serum (rHEVgt3‐HIS; Institute for Novel and Emerging Infectious Diseases, Friedrich‐Loeffler‐Institut, Germany) in a 1:500 dilution at 4°C overnight (approx. 18 h). Then, samples were washed in PBS and incubated for 30 min at RT with biotinylated goat anti‐rabbit IgG secondary antibody (Vector Laboratories, Burlingame, CA) diluted 1:200 in TBS containing 10% normal goat serum. After washing with TBS (pH 7.6), all tissue sections were finally incubated with ABC complex (Vectastain ABC Elite Kit; Vector Laboratories) for 1 h at RT and in the dark. The sections were finally washed in PBS and incubated with a chromogen solution (Nova RED substrate kit, Vector Laboratories, Burlingame, CA) and counterstained with haematoxylin.

As positive controls, liver and hepatic lymph node samples from a pig experimentally infected with HEV genotype 3 were used, confirmed by real‐time RT‐PCR and IHC (samples from the study of Schlosser et al., 2014). As negative controls, wild boar tissues without evidence of HEV‐RNA in serum or randomly selected tissues were used. Moreover, rabbit non‐immune sera (Vector Laboratories, Burlingame, CA) were used in place of specific primary antibody as additional negative controls.

The evaluation of the immunohistochemical examination in the organs selected was performed for the presence of target cells to anti‐HEV antibody, classifying the organs as without staining (–), and with scarce (+), moderate (++) or high (+++) number of immunolabelled cells.

### 2.4 Statistical analysis

The prevalence of HEV was determined by the coefficient of positive animals/total animals tested, using two‐sided exact binomial 95% confidence intervals (95%CI).

### 2.5 Ethical and biosafety aspects of research

The animals were legally hunted under Spanish and EU legislation and all the hunters had hunting licenses. Professional personnel collected the blood and tissue samples from hunter‐harvested wild boar during the hunting seasons. This study did not involve purposeful killing of animals, since all the samples were collected as part of routine procedures before the design of this study. So, no ethical approval was deemed necessary in compliance with the Ethical Principles in Animal Research. Protocols, amendments and other resources were followed according to the guidelines approved by each Autonomous government following the R.D.1201/2005 and R.D.53/2013 of the Spanish Ministry, which establish the basic normative for the protection of animals used for scientific purposes.

Biological samples were treated as infectious material according to Biosafety Level 2, following the specific management, analysis and elimination measures indicated in the Waste and Contaminated Soils Act 22/2011 of 28 July of the Government of Spain.

## 3 RESULTS

Of the 194 male wild boars included in the study, three were discarded due to the poor quality of their serum and/or because there was not enough quantity of sample for RT‐PCR. Of the 191 male wild boars available, four of them (2.09%; 95%CI: 0.82–5.26) showed detectable HEV RNA in serum. The HEV RNA sequences were detected in three of the four animals. These sequences were registered in GenBank with accession numbers OM525661 to OM525663. Sequencing of ORF1 region allowed designation of the three strains as genotype 3f.

**FIGURE 1:**
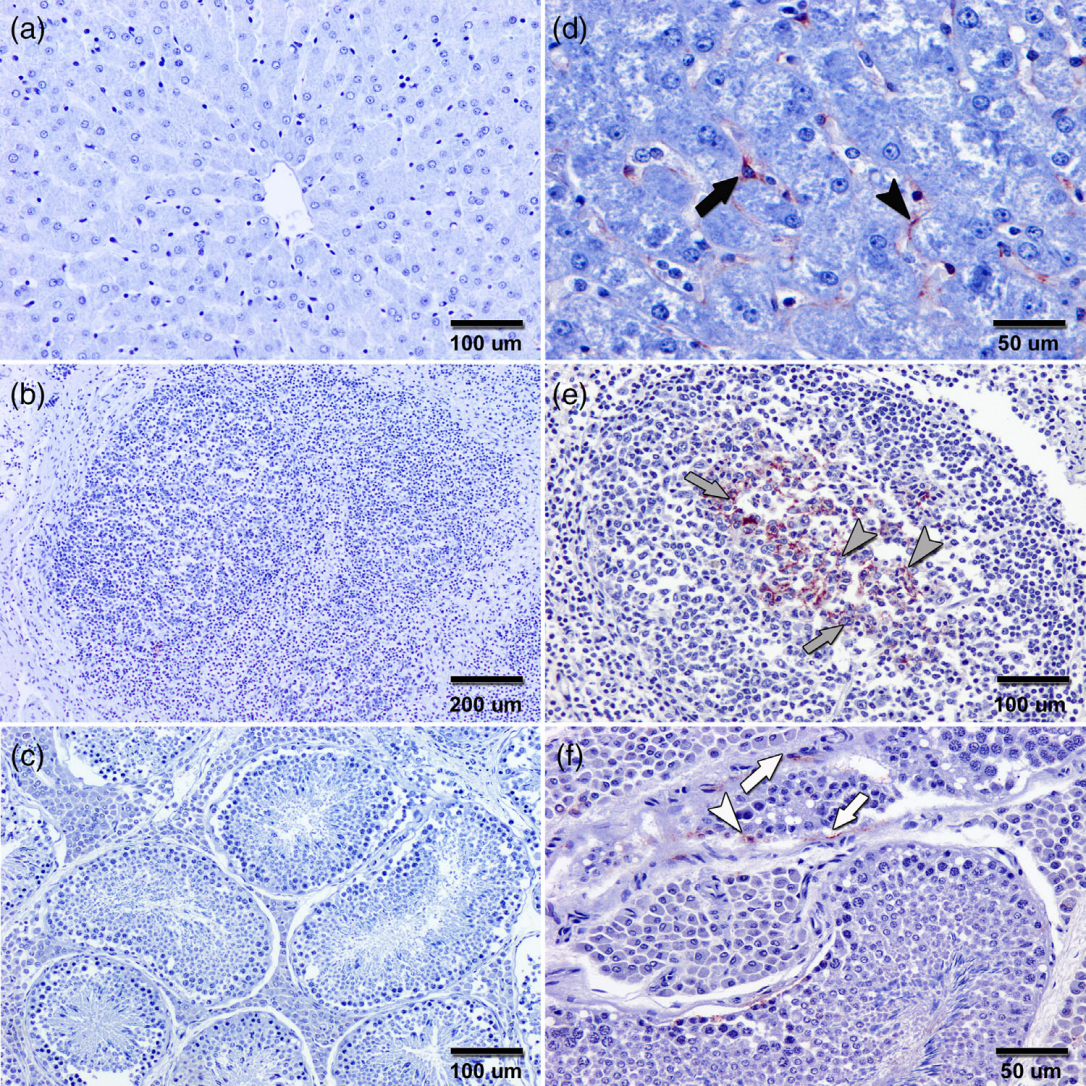
Representative photomicrographs of tissue sections from wild boar naturally infected with hepatitis E virus (HEV), which was detected by real‐time RT‐PCR in serum. No immunohistochemical signals were observed in the liver (a), hepatic lymph node (b) and testis (c) of wild boar in which HEV RNA was not detected in these tissues. Meanwhile, in the naturally infected wild boar with HEV RNA detected by real‐time RT‐PCR in the different tissues, HEV antigen was also found in hepatic tissue (d), mainly in Kupffer cells (black arrow) and liver sinusoidal endothelial cells (black arrowhead). Likewise, in this animal, HEV was observed in macrophages (grey arrows) and cells with dendritic morphology (grey arrowheads) of the hepatic lymph node (e) and lightly in fibroblasts (white arrows) and some Sertoli cells (white arrowhead) of testis (f). Immunohistochemistry (IHC) with the avidin‐biotin‐peroxidase complex (ABC) method counterstained with haematoxylin

**FIGURE 2:**
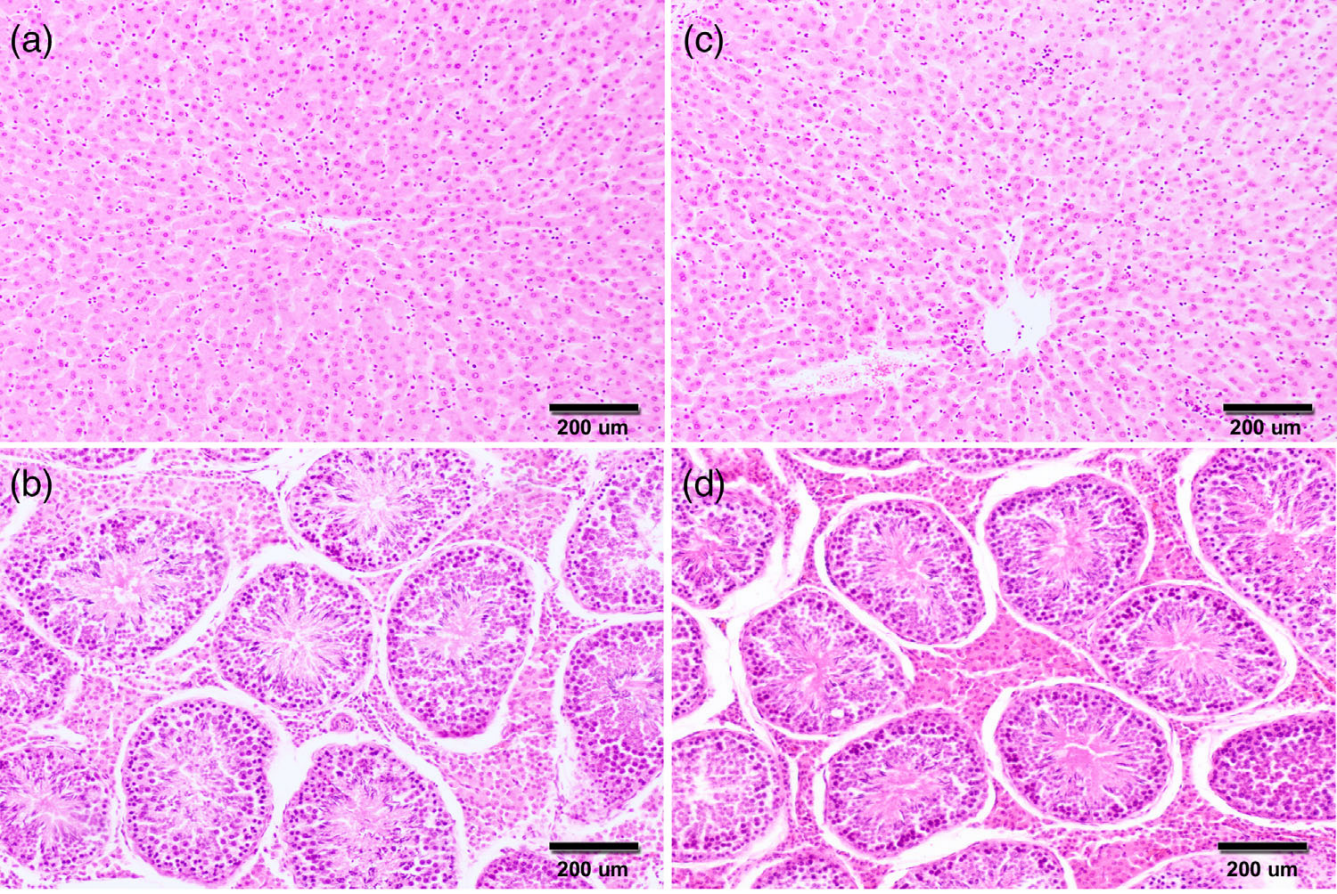
Histopathological study in wild boar naturally infected with hepatitis E virus (HEV). None of the two animals, either HEV‐negative by real‐time RT‐PCR in liver and testis (a and b) or HEV‐positive in liver and testis (c and d, respectively), showed histopathological lesions in these organs. Haematoxylin and eosin (HE) staining

None of the 187 boars with viral RNA‐negative in serum showed positive HEV‐RNA in the analysed tissues (liver, hepatic lymph node and testis). No immunohistochemical signals using a rabbit anti‐HEV gt3 hyperimmune serum (rHEVgt3‐HIS) were observed in these tissues of viral RNA‐negative wild boars in serum and tissues (Figure 1a–c). On the other hand, Table 1 shows the results of HEV in tissues of those boars with positive HEV in serum, being consistent the molecular and immunohistochemical analyses. Of the 4 male wild boars positive to HEV‐RNA in serum, IHC located viral antigens in the hepatic tissue of the 3 animals with HEV detected by RT‐PCR. HEV antigen in hepatic tissue was found mainly in the Kupffer cells and liver sinusoidal endothelial cells (Figure 1d). HEV antigen was also stained in granular patterns in the cytoplasm of macrophages and cells with dendritic morphology within the lymphoid follicles of hepatic lymph nodes from 2 of the 3 animals with HEV detected by RT‐PCR (Figure 1e). Moreover, HEV‐specific labelling appeared as evenly and lightly distributed in a small number of fibroblasts and some Sertoli cells of the HEV‐positive testicle by RT‐PCR (Figure 1f). On the other hand, histopathological examination of the tissues of wild boar naturally infected with HEV did not present any remarkable pathological change (Figure 2).

**TABLE 1:** . Characteristics of HEV‐positive wild boars and evaluation of HEV detection in serum and tissues

Age	HEV‐RNA serum (UI/ml)	HEV‐RNA in liver (Ct)	IHC in liver	HEV‐RNA in hepatic lymph node (Ct)	IHC in hepatic lymph node	HEV‐RNA in testis (Ct)	IHC in testis
Adult	**Positive** 5796	**Positive** 35.32	+	**Positive** 37.15	–	Negative	–
Subadult	**Positive** 1097	**Positive** 34.80	++	Negative	–	Negative	–
Adult	**Positive** 184	Negative	–	**Positive** 31.35	++	Negative	–
Adult	**Positive** 367,491	**Positive** 28.70	+++	**Positive** 32.04	++	**Positive** 32.50	+

*Note*: Immunohistochemical examination for anti‐HEV antibody was classified as absence of staining (–), scarce (+), moderate (++) and high (+++) number of immunolabelled cells.

HEV: hepatitis E virus; RNA: ribonucleic acid; IHC: immunohistochemistry.

## 4 DISCUSSION

During the acute phase of infection in humans, HEV can be detected in various body fluids, such as breast milk (Rivero‐Juarez et al., 2016), saliva (Rivero‐Juarez et al., 2018), urine (Marion et al., 2019) and cerebrospinal fluid (van Eijk et al., 2017). The presence of the virus in body fluids has a significant impact in terms of transmission, diagnosis or extrahepatic manifestations during the acute phase of infection. Several studies have isolated HEV in the genitourinary tract of humans and animals, which could have important clinical and epidemiological implications (Geng et al., 2016; Huang et al., 2018; Li et al., 2019).

A study carried out in China in infertile men showed a prevalence of HEV RNA genotype 4 in semen of 28.1%, with comparable viral titres in urine (Huang et al., 2018). In the same way, a study conducted in China including 26 semen samples collected from pigs found the presence of HEV RNA genotype 4 in one individual (3.3%) (Li et al., 2019). However, in several studies performed in Germany, the presence of HEV‐RNA genotype 3 was not demonstrated in semen samples (*n* = 87) of infertile men (Horvatits et al., 2020) or in patients with acute self‐limiting hepatitis E (Horvatits et al., 2021), neither in testis of pigs intravenously infected with HEV genotype 3 (Horvatits et al., 2021). Nevertheless, genotype 3c was detected in the ejaculate of chronically immunosuppressed HEV‐infected men, even more than nine months after clearance of HEV viraemia (Horvatits et al., 2021). The difference in the results of these studies could suggest a relationship between the HEV genotype and the ability to produce testicular involvement and infertility. However, another study conducted in China did not confirm the presence of HEV‐RNA in semen samples of 1183 infertile men diagnosed at the Department of Reproductive Medicine Center, Peking University (Wang et al., 2020). In the present study, the presence of HEV RNA in sperm could not be evaluated due to the logistical characteristics of the sampling, but the virus was detected by RT‐PCR and IHC in the testis of one of the wild boars naturally infected with genotype 3. These results evidence that HEV genotype 3 can be also detected in the male genital tract of swine, which could have important implications in the transmission of the virus by natural reproduction in swine. Therefore, our results give reasons to reconsider the protocols of HEV diagnosis in semen intended for artificial insemination used in pig breeding to be screened for this virus.

The presence of HEV in testis of swine could also have important clinical implications in terms of fertility. The experimental infection of two rhesus macaques (*Macaca mulatta*) with HEV genotype 4 demonstrated the presence of virus in epididymis and testis, mainly in spermatogonia, associated to a destruction of the blood‐testis barrier and the seminiferous epithelium (Huang et al., 2018). These lesions, together with the death of germ cells, have also been observed in BALB/c mice experimentally infected with HEV genotype 4, leading to a decrease in the sperm count, the presence of abnormalities in them and an increased necrospermia (Situ et al., 2020). All this gave rise to a transient infertility in the mice, which could partially be recovered after the complete elimination of HEV, whose permanence was longer in testis than in blood or faeces (42 vs. 28 days post‐infection). Likewise, in animal models as Mongolian gerbils (*Meriones unguiculatus*), genotype 4 has also shown to induce molecular and structural changes in testis, damaging the blood‐testis barrier (Soomro et al., 2017). In contrast, evidence of testicular damage in wild boars naturally infected with genotype 3 was not found in our study, even though HEV was detected in this organ by RT‐PCR and IHC. Consequently, HEV genotype 3 infection does not seem to have a negative impact in fertility of male swine, as has been observed in female, with histopathological differences in the genitourinary tract between HEV genotypes. Similarly, it has been shown that HEV genotype 4 can replicate in the ovaries and promote apoptosis of oocytes (An et al., 2017) and genotype 1 has a high tropism for the placenta and decidua, inducing tissue apoptosis and necrosis (Gouilly et al., 2018), whereas genotype 3 showed a lack of tropism and induction of tissue damage in both structures.

This pilot study has some limitations that should be considered. The number of individuals with HEV infection included in our study was low. Second, the animals included in the study were naturally infected, so it was not possible to assess the timing of the presence of HEV in the testis. In this type of infections, which reliably represent what happens in real conditions, the viral load to which individuals are exposed is usually lower than in an experimental infection, and the results, viral distribution and tissue damage described cannot be fully compared in both study models.

In conclusion, our results have demonstrated the presence of HEV genotype 3 in testis of naturally infected wild boars, although no association with tissue damage has been evidenced. This study does not allow to rule out semen as a possible source of viral transmission in suids. Future experimental studies are necessary to evaluate the impact of HEV genotype 3 on fertility and the possibility of transmission through sexual contact.

## AUTHOR CONTRIBUTIONS

Dr. Rivero‐Juarez had full access to all of the data in the study and takes responsibility for the integrity of the data and the accuracy of the data analysis.

Study concept and design: MARM and ARJ. Sample collection: MARM, SJR, IAR, DJM. Sample collection and procedures: MF, JCG, PLL, MARM, CF and ME. Analysis and interpretation of the data: MARM, MF and ARJ. Drafting of the manuscript: MARM, ARJ. Critical revision of the manuscript for important intellectual content: All authors. Statistical analysis: MARM and ARJ. Obtained funding: MARM, AR and ARJ.

## CONFLICT OF INTEREST

The authors have declared no conflict of interest.

## ETHICS STATEMENT

This study did not involve purposeful killing of animals. Professional personnel collected blood and liver samples mostly from hunted‐harvested wild boar during the hunting season. These animals were legally hunted under Spanish and EU legislation and all hunters had hunting licenses. No ethical approval was deemed necessary; all collection of samples was performed for routine procedures before the design of this study in compliance with the Ethical Principles in Animal Research. Thus, blood or liver samples were not collected specifically for this study. Protocols, amendments and other resources were all done according to the guidelines approved by each Autonomous government following the R.D.1201/2005 of the Ministry of Presidency of Spain.

## Data Availability

All data generated or analysed during the study are included in this published article. The datasets used and/or analysed during the present research project are available from the corresponding author on reasonable request. Sequences are available in GenBank under accession numbers OM525661, OM525662 and OM525663.

## References

[tbed14682-bib-0001] An, J. , Liu, T. , She, R. , Wu, Q. , Tian, J. , Shi, R. , Hao, W. , Ren, X. , Yang, Y. , Lu, Y. , Yang, Y. , & Wu, Y. (2017). Replication of hepatitis E virus in the ovary and promotion of oocyte apoptosis in rabbits infected with HEV‐4. Oncotarget, 9, 4475–4484. 10.18632/oncotarget.23381 29435117 PMC5796988

[tbed14682-bib-0002] Arenas‐Montes, A. , García‐Bocanegra, I. , Paniagua, J. , Franco, J. J. , Miró, F. , Fernández‐Morente, M. , & Arenas, A. (2013). Blood sampling by puncture in the cavernous sinus from hunted wild boar. European Journal of Wildlife Research, 59, 299–303. 10.1007/s10344-013-0701-3

[tbed14682-bib-0003] Denner, J. (2019). Hepatitis E virus (HEV) – The future. Viruses, 11, 251. 10.3390/v11030251 30871152 PMC6466233

[tbed14682-bib-0004] Domanović, D. , Tedder, R. , Blümel, J. , Zaaijer, H. , Gallian, P. , Niederhauser, C. , Sauleda Oliveras, S. , O'Riordan, J. , Boland, F. , Harritshøj, L. , Nascimento, M. S. J. , Ciccaglione, A. R. , Politis, C. , Adlhoch, C. , Flan, B. , Oualikene‐Gonin, W. , Rautmann, G. , Strengers, P. , & Hewitt, P. (2017). Hepatitis E and blood donation safety in selected European countries: A shift to screening? Eurosurveillance, 22, 30514. 10.2807/1560-7917.ES.2017.22.16.30514 28449730 PMC5404480

[tbed14682-bib-0005] Faber, M. , Askar, M. , & Stark, K. (2018). Case‐control study on risk factors for acute hepatitis E in Germany, 2012 to 2014. Eurosurveillance, 23, 17–00469. 10.2807/1560-7917.ES.2018.23.19.17-00469 PMC5954605 29766841

[tbed14682-bib-0006] Frías, M. , López‐López, P. , Zafra, I. , Caballero‐Gómez, J. , Machuca, I. , Camacho, Á. , Risalde, M. A. , Rivero‐Juárez, A. , & Rivero, A. (2021). Development and clinical validation of a pangenotypic PCR‐based assay for the detection and quantification of hepatitis E virus (*Orthohepevirus* A Genus). Journal of Clinical Microbiology, 59, e02075–20. 10.1128/JCM.02075-20 33148702 PMC8111163

[tbed14682-bib-0007] Geng, Y. , Zhao, C. , Huang, W. , Harrison, T. J. , Zhang, H. , Geng, K. , & Wang, Y. (2016). Detection and assessment of infectivity of hepatitis E virus in urine. Journal of Hepatology, 64, 37–43. 10.1016/j.jhep.2015.08.034 26362822

[tbed14682-bib-0008] Gouilly, J. , Chen, Q. , Siewiera, J. , Cartron, G. , Levy, C. , Dubois, M. , Al‐Daccak, R. , Izopet, J. , Jabrane‐Ferrat, N. , & El Costa, H. (2018). Genotype specific pathogenicity of hepatitis E virus at the human maternal‐fetal interface. Nature Communications, 9, 4748. 10.1038/s41467-018-07200-2 PMC6232144 30420629

[tbed14682-bib-0009] Horvatits, T. , & Pischke, S. (2018). Extrahepatic manifestations and HEV, the genotype matters. EBioMedicine, 36, 3–4. 10.1016/j.ebiom.2018.09.004 30213536 PMC6197509

[tbed14682-bib-0010] Horvatits, T. , Varwig‐Janssen, D. , Zur Wiesch, J. S. , Lübke, R. , Reucher, S. , Frerk, S. , Addo, M. M. , Schulze Zur Wiesch, J. , Lohse, A. W. , Lütgehetmann, M. , & Pischke, S. (2020). No link between male infertility and HEV genotype 3 infection. Gut, 69, 1150–1151. 10.1136/gutjnl-2019-319027 31118248

[tbed14682-bib-0011] Horvatits, T. , Wißmann, J. E. , Johne, R. , Groschup, M. H. , Gadicherla, A. K. , Schulze Zur Wiesch, J. , Eiden, M. , Todt, D. , Reimer, R. , Dähnert, L. , Schöbel, A. , Horvatits, K. , Lübke, R. , Wolschke, C. , Ayuk, F. , Rybczynski, M. , Lohse, A. W. , Addo, M. M. , Herker, E. , … Pischke, S. (2021). Hepatitis E virus persists in the ejaculate of chronically infected men. Journal of Hepatology, 75, 55–63. 10.1016/j.jhep.2020.12.030 33484776

[tbed14682-bib-0012] Huang, F. , Long, F. , Yu, W. , Situ, J. , Fu, L. , He, Z. , Dong, H. , Yang, C. , Li, Y. , Yang, F. , & Wei, D. (2018). High prevalence of hepatitis E virus in semen of infertile male and causes testis damage. Gut, 67, 1199–1201. 10.1136/gutjnl-2017-314884 28860349

[tbed14682-bib-0013] Johne, R. , Plenge‐Bönig, A. , Hess, M. , Ulrich, R. G. , Reetz, J. , & Schielke, A. (2010). Detection of a novel hepatitis E‐like virus in faeces of wild rats using a nested broad‐spectrum RT‐PCR. Journal of General Virology, 91, 750–758. 10.1099/vir.0.016584-0 19889929

[tbed14682-bib-0014] Li, H. , Wu, J. , Sheng, Y. , Lu, Q. , Liu, B. , Chen, Y. , Sun, Y. , Zhou, E. M. , & Zhao, Q. (2019). Prevalence of hepatitis E virus (HEV) infection in various pig farms from Shaanxi Province, China: First detection of HEV RNA in pig semen. Transboundary and Emerging Diseases, 66, 72–82. 10.1111/tbed.12966 30043495

[tbed14682-bib-0015] Marion, O. , Capelli, N. , Lhomme, S. , Dubois, M. , Pucelle, M. , Abravanel, F. , Kamar, N. , & Izopet, J. (2019). Hepatitis E virus genotype 3 and capsid protein in the blood and urine of immunocompromised patients. Journal of Infection, 78, 232–240. 30659856 10.1016/j.jinf.2019.01.004

[tbed14682-bib-0016] McPherson, S. , Elsharkawy, A. M. , Ankcorn, M. , Ijaz, S. , Powell, J. , Rowe, I. , Tedder, R. , & Andrews, P. A. (2018). Summary of the British Transplantation Society UK Guidelines for Hepatitis E and Solid Organ Transplantation. Transplantation, 102, 15–20. 10.1016/j.jinf.2019.01.004 28795981

[tbed14682-bib-0017] Meng, X. J. (2013). Zoonotic and foodborne transmission of hepatitis E virus. Seminars in Liver Disease, 33, 41–49. 10.1055/s-0033-1338113 23564388

[tbed14682-bib-0018] Nimgaonkar, I. , Ding, Q. , Schwartz, R. E. , & Ploss, A. (2018). Hepatitis E virus: Advances and challenges. Nature Reviews Gastroenterology & Hepatology, 15, 96–110. 29162935 10.1038/nrgastro.2017.150 PMC11329273

[tbed14682-bib-0019] Purcell, R. H. , & Emerson, S. U. (2008). Hepatitis E: An emerging awareness of an old disease. Journal of Hepatology, 48, 494–503. 10.1038/nrgastro.2017.150 18192058

[tbed14682-bib-0020] Purdy, M. A. , Drexler, J. F. , Meng, X. , Norder, H. , Okamoto, H. , Van der Poel, W. H. M. , Reuter, G. , de Souza, W. M. , Ulrich, R. G. , & Smith, D. B. (2022). ICTV virus taxonomy profile: Hepeviridae. Journal of General Virology, 98(11), 2645–2646. 10.1099/jgv.0.000940 PMC5718254 29022866

[tbed14682-bib-0021] Risalde, M. A. , Rivero‐Juárez, A. , Romero‐Palomo, F. , Frías, M. , López‐López, P. , Cano‐Terriza, D. , García‐Bocanegra, I. , Jiménez‐Ruíz, S. , Camacho, Á. , Machuca, I. , Gomez‐Villamandos, J. C. , & Rivero, A. (2017). Persistence of hepatitis E virus in the liver of non‐viremic naturally infected wild boar. PLoS One, 12, e0186858. 10.1371/journal.pone.0186858 29117209 PMC5678868

[tbed14682-bib-0022] Rivero‐Juárez, A. , Aguilera, A. , Avellón, A. , García‐Deltoro, M. , García, F. , Gortazar, C. , Granados, R. , Macías, J. , Merchante, N. , Oteo, J. A. , Pérez‐Gracia, M. T. , Pineda, J. A. , Rivero, A. , Rodriguez‐Lazaro, D. , Téllez, F. , & Morano‐Amado, L. E. (2020). Executive summary: Consensus document of the diagnosis, management and prevention of infection with the hepatitis E virus: Study Group for Viral Hepatitis (GEHEP) of the Spanish Society of Infectious Diseases and Clinical Microbiology (SEIMC). Enfermedades Infecciosas y Microbiologia Clinica (English ed.), 38, 28–32. 10.1016/j.eimce.2018.06.014 30072282

[tbed14682-bib-0023] Rivero‐Juarez, A. , Frias, M. , Lopez‐Lopez, P. , Martinez‐Peinado, A. , Risalde, M. Á. , Brieva, T. , Machuca, I. , Camacho, Á. , García‐Bocanegra, I. , Gomez‐Villamandos, J. C. , & Rivero, A. (2018). Detection of hepatitis E virus RNA in saliva for diagnosis of acute infection. Zoonoses Public Health, 65, 584–588. 10.1111/zph.12472 29659194

[tbed14682-bib-0024] Rivero‐Juarez, A. , Frias, M. , Rodriguez‐Cano, D. , Cuenca‐López, F. , & Rivero, A. (2016). Isolation of hepatitis E virus from breast milk during acute infection. Clinical Infectious Diseases, 62, 1464. 10.1093/cid/ciw186 27025819

[tbed14682-bib-0025] Schlosser, J. , Eiden, M. , Vina‐Rodriguez, A. , Fast, C. , Dremsek, P. , Lange, E. , Ulrich, R. G. , & Groschup, M. H. (2014). Natural and experimental hepatitis E virus genotype 3‐infection in European wild boar is transmissible to domestic pigs. Veterinary Research, 45, 121. 10.1186/s13567-014-0121-8 25421429 PMC4243386

[tbed14682-bib-0026] Situ, J. , Wang, W. , Long, F. , Yang, W. , Yang, C. , Wei, D. , Yu, W. , & Huang, F. (2020). Hepatitis E viral infection causes testicular damage in mice. Virology, 541, 150–159. 10.1016/j.virol.2019.12.009 32056713

[tbed14682-bib-0027] Soomro, M. H. , Shi, R. , She, R. , Yang, Y. , Wang, T. , Wu, Q. , Li, H. , & Hao, W. (2017). Molecular and structural changes related to hepatitis E virus antigen and its expression in testis inducing apoptosis in Mongolian gerbil model. Journal of Viral Hepatitis, 24, 696–707. 10.1111/jvh.12690 28182318

[tbed14682-bib-0028] Syed, S. F. , Zhao, Q. , Umer, M. , Alagawany, M. , Ujjan, I. A. , Soomro, F. , Bangulzai, N. , Baloch, A. H. , El‐Hack, M. A. , Zhou, E. M. , & Arain, M. A. (2018). Past, present and future of hepatitis E virus infection: Zoonotic perspectives. Microbial Pathogenesis, 119, 103–108. 10.1016/j.micpath.2018.03.051 29621564

[tbed14682-bib-0029] van Eijk, J. J. J. , Dalton, H. R. , Ripellino, P. , Madden, R. G. , Jones, C. , Fritz, M. , Gobbi, C. , Melli, G. , Pasi, E. , Herrod, J. , Lissmann, R. F. , Ashraf, H. H. , Abdelrahim, M. , Masri, O. A. B. A. L. , Fraga, M. , Benninger, D. , Kuntzer, T. , Aubert, V. , Sahli, R. , … van Engelen, B. G. M. (2017). Clinical phenotype and outcome of hepatitis E virus‐associated neuralgic amyotrophy. Neurology, 89, 909–917. 10.1212/WNL.0000000000004297 28768846

[tbed14682-bib-0030] Wang, L. , Teng, J. L. , Lau, S. K. , Sridhar, S. , Fu, H. , Gong, W. , Li, M. , Xu, Q. , He, Y. , Zhuang, H. , Woo, P. C. , & Wang, L. (2019). Transmission of a novel genotype of hepatitis E virus from Bactrian camels to cynomolgus macaques. Journal of Virology, 93, e02014–18. 10.1128/JVI.02014-18 30700602 PMC6430554

[tbed14682-bib-0031] Wang, L. , Zhang, Z. , Shu, J. , Zhang, H. , Yang, Y. , Liang, Z. , Liang, Z. , He, Q. , Huang, W. , Wang, Y. , Zhuang, H. , Jiang, H. , & Wang, L. (2020). Absence of hepatitis E virus RNA in semen samples of infertile male in China. Gut, 69, 1363–1364. 10.1136/gutjnl-2019-319234 31217236

[tbed14682-bib-0032] World Health Organization (WHO) (2017). Global hepatitis report, 2017. Retrieved from: https://www.who.int/hepatitis/publications/global‐hepatitis‐report2017/en/

